# Visualizing Fluid Flows via Regularized Optimal Mass Transport with Applications to Neuroscience

**DOI:** 10.1007/s10915-023-02337-9

**Published:** 2023-09-19

**Authors:** Xinan Chen, Anh Phong Tran, Rena Elkin, Helene Benveniste, Allen R. Tannenbaum

**Affiliations:** 1 Department of Medical Physics, Memorial Sloan Kettering Cancer Center, 1275 York Ave, New York, NY 10065, USA; 2 Department of Anesthesiology, Yale School of Medicine, 333 Cedar St, New Haven, CT 06510, USA; 3 Department of Computer Science, Stony Brook University, 100 Nicolls Rd, Stony Brook, NY 11794, USA; 4 Department of Applied Mathematics and Statistics, Stony Brook University, 100 Nicolls Rd, Stony Brook, NY 11794, USA

**Keywords:** Regularized optimal mass transport, Fluid dynamics, Computational framework, 35A15, 65D18, 76R99

## Abstract

The regularized optimal mass transport (rOMT) problem adds a diffusion term to the continuity equation in the original dynamic formulation of the optimal mass transport (OMT) problem proposed by Benamou and Brenier. We show that the rOMT model serves as a powerful tool in computational fluid dynamics for visualizing fluid flows in the glymphatic system. In the present work, we describe how to modify the previous numerical method for efficient implementation, resulting in a significant reduction in computational runtime. Numerical results applied to synthetic and real-data are provided.

## 1 Introduction

Optimal mass transport (OMT) treats the problem of optimally transporting a mass distribution from one configuration to another via the minimization of a given cost function. The OMT problem was first posed by Monge in 1781 in the context of the transportation of debris [24]. This formulation was later given a modern relaxed formulation by Kantorovich [17]. Benamou and Brenier [1] reformulated OMT into a computational fluid dynamics (CFD) framework. In recent times, OMT theory has received extensive research attention with rich applications in machine learning [21, 28], image processing/registration [11, 12, 14], network theory [4], and biomedical science [33].

The model employed in this work is based on the CFD approach proposed by Benamou and Brenier [1]. Here OMT is formulated as an energy minimization problem with a partial differential equation (continuity) constraint. The continuity equation in the original version only involves advection. Our implementation includes an additional diffusion term that is of importance in our studies of glymphatic flows to analyze these two major transport motions [3, 22] in the brain. See more discussion of this added diffusion term in Sect. 4. This leads to the present modified formulation, which is referred as the *regularized* optimal mass transport (rOMT) problem. In addition to visualizing glymphatic flows, this type of model appears in many contexts including the Schrödinger bridge and entropic regularization, from which the popular Sinkhorn algorithm is derived [7–9].

The rOMT model is formally described as follows. Given two non-negative density/mass functions $\rho_{0}(x)$ and $\rho_{1}(x)$ defined on spatial domain $\Omega \subseteq R^{3}$ with equal total mass $\int_{\Omega }\;\rho_{0}(x)dx=\int_{\Omega }\;\rho_{1}(x)dx$, we consider the following optimization problem:

(1) $$\underset{\rho ,v}{inf}\;\int_{0}^{1}\int_{\Omega }\rho (t,\;x)∥v(t,\;x){∥}^{2}dxdt$$

subject to

(2a) $$\frac{\partial \rho }{\partial t}+\nabla \cdot (\rho v)=\sigma \Delta \rho ,$$

(2b) $$\rho (0,x)=\rho_{0}(x),\;\rho (1,x)=\rho_{1}(x)$$

Where ρ(t,x):[0,1]×Ω→R and $v\left(t,\;x\right)\;:[0,\;1]\;\times \;\Omega \;\to \;R^{3}$ are the time-dependent density/mass function and velocity field, respectively, ∥⋅∥ represents the Euclidean norm, and σ>0 is the constant diffusion coefficient. Equation 2a is the *advection–diffusion equation* in fluid dynamics. It may be regarded as the Fokker–Planck equation corresponding to an underlying stochastic process modelled by a stochastic differential equation [13]. If we set σ=0, one can recover the regular OMT problem proposed by Benamou and Brenier [1]. By adding the non-negative diffusion term σΔρ to the continuity equation, we include both motions, advection and diffusion, into the dynamics of the system. The system Eqs. 1–2b solves for the optimal interpolation ρ(t,x) between the initial and final density/mass distributions, $\rho_{0}(x)$ and $\rho_{1}(x)$, and for the optimal velocity field v(t,x) which transports $\rho_{0}(x)$ into $\rho_{1}(x)$ in such a way that the total kinetic energy is minimized and the dynamics follow the advection–diffusion equation. Continuing the work of the numerical method in [6, 10, 18], we report a significant reduction in runtime by about 91% resulting from improvements of previous code.

Some of the primary applications of the present work are concerned with fluid and solute flows in the brain, and in particular, the glymphatic system. The latter is a waste clearance network in the central nervous system that is mainly active during sleep and with certain anesthetics. Many neurodegenerative diseases, such as Alzheimer’s and Parkinson’s, are believed to be related to the impairment of the glymphatic system. The glymphatic transport network has received enormous attention and efforts of a number of researchers to understand the fluid behaviors in the waste disposal process in the brain [2, 16, 25, 26, 31]. The rOMT formulation described in the present work is highly relevant to analyzing glymphatic data due to the inclusion of both advection and diffusion terms in the partial differential equation. In addition to solving the rOMT problem, we use Lagrangian coordinates for the rOMT model, which is especially useful for visualization of the time trajectories of the transport.

## 2 Material and Methods

This section outlines the numerical method of solving the rOMT problem, Eqs. 1–2b, and a post-processing Lagrangian method for practical purposes of tracing particles and visualizing fluid flows.

### 2.1 Numerical Solution of rOMT

The developed method is based on the assumption that the intensity of observed dynamic contrast enhanced MRI (DCE-MRI) data is proportional to the density/mass function in the rOMT model, and thus we treat the image intensity as the concentration of the tracer molecules *in vivo*. Suppose we are given the observed initial and final images, $\rho_{0}^{\mathrm{obs}}(x)$ and $\rho_{1}^{obs}(x)$. Notice that these observed images are in a noisy state. By posing a fixed end-point condition $\rho (1,\;x)=\rho_{1}^{\mathrm{obs}}(x)$, we may be over-matching the image noise which interferes with the real dynamics. In consideration of that, instead of implementing a fixed end-point condition, we use a free end-point of the advection–diffusion process, which is realized by adding a fitting term ${∥\rho (1,\;x)-\rho_{1}^{obs}(x)∥}^{2}$ into the cost function and removing ρ from the optimized variables. This free end-point version of the rOMT problem for applications in noisy (e.g., DCE-MRI) data may be expressed as

(3) $$\underset{v}{inf}\;\int_{0}^{1}\int_{\Omega }\rho (t,\;x)∥v(t,\;x){∥}^{2}dxdt+\beta {∥\rho (1,\;x)-\rho_{1}^{obs}(x)∥}^{2}$$

subject to

(4a) $$\frac{\partial \rho }{\partial t}+\nabla \cdot \left(\rho v\right)=\sigma \Delta \rho ,$$

(4b) $$\rho (0,\;x)=\rho_{0}^{obs}(x)$$

where β is the weighting parameter balancing between minimizing the kinetic energy and matching the final image. By adding the $L^{2}$ approximation term in the cost function, our numerical method is given an unbalanced flavor in that the total mass of the initial and final images need not be exactly conserved. Given successive images $\rho_{0}^{\mathrm{obs}},\rho_{1}^{\mathrm{obs}},\ldots ,\rho_{p-1}^{\mathrm{obs}}$ where p>2 and $p\in N^{+}$, this method can be recursively run between adjacent images to guide the prolonged dynamic solution.

Note that in Benamou and Brenier’s work [1], they numerically solved the OMT problem by changing the variables from ρ and v to ρ and momentum P=ρv, which consequently gives a linear constraint. However, in our work, we need to use the variable v rather than P due to the fact that ρ may be very small or even 0 in the DCE-MRI data when the signal is weak. With the variable P, the integrand of the first term in the cost function Eq. 3 becomes $\frac{∥P(t,x){∥}^{2}}{\rho (t,x)}$, which will go to infinity as ρ approaches 0.

Next, a 3D version of the algorithm is detailed. Note that the proposed workflow also works for 2D problems with simple modifications. The spacial domain Ω is discretized into a cell-centered uniform grid of size $n_{x}\times n_{y}\times n_{z}$ and the time space is divided into m equal intervals. Let $k_{s}$ and $k_{t}$ be the volume of each spatial voxel and the length of each time interval, respectively. With $t_{i}=i\cdot k_{t}$ for i=0,…,m denoting the m+1 discrete time steps, we have discrete interpolations and velocity fields, $\rho =\left[\rho_{1};\ldots ;\rho_{m}\right]$ and $v=\left[v_{0};\ldots ;v_{m-1}\right]$ where $\rho_{i}$ is the interpolated image at $t=t_{i}$ and $v_{i}$ is the velocity field transporting $\rho_{i}$ to $\rho_{i+1}$. Note that a bold font is used to denote discretized flattened vectors to differentiate from continuous functions. For example, ρ is a vector of size mn×1 and v is of size 3mn×1 where $n=n_{x}n_{y}n_{z}$ is the total number of voxels.

The cost function Eq. 3 can be approximated with

(5) $$F\left(v\right)\approx k_{s}k_{t}\rho^{T}M\left(v⊙v\right)+{∥\rho_{m}-\rho_{1}^{obs}∥}^{2},$$

where $M=I_{m}⊗\left[I_{n}\left|I_{n}\right|I_{n}\right]$. Here ⊗ is the Kronecker product; ⊙ is the Hadamard product; $I_{i}$ is the i×i identity matrix; [⋅∣⋅] denotes forming block matrices.

**Fig. 1:**
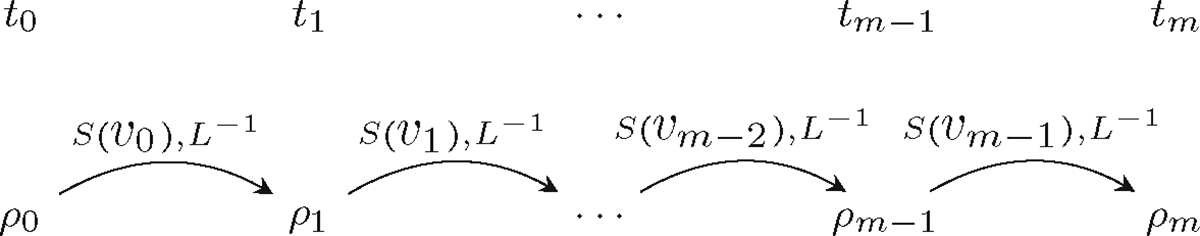
Numerical Pipeline of rOMT: From $t_{i}$ to $t_{i+1}$, the interpolated image $\rho_{i}$ is firstly advected via the velocity field $v_{i}$ by applying averaging matrix $S\left(v_{i}\right)$ and is next diffused by applying matrix $L^{-1}$ for i=0,…,m−1

An operator splitting technique is employed to separate the transport process into an advective and a diffusive step. From $t_{i}$ to $t_{i+1}$, in the firstly advective step, a particle-in-cell method is used to re-allocate transported mass to its nearest cell centers:$\rho_{i+1}^{adv}=S\left(v_{i}\right)\rho_{i}$, where $S\left(v_{i}\right)$ is the interpolation matrix after the movement by velocity field $v_{i}$. In the secondly diffusive step, we use Euler backwards scheme to ensure stability: $\frac{1}{k_{t}}\left(\rho_{i+1}-\rho_{i+1}^{adv}\right)=Q\rho_{i+1}$ where Q is the discretization matrix of the diffusive operator σΔ on a cell-centered grid. Combining the two steps together, we get the discretized advection–diffusion Eq. 4a:

(6) $$\rho_{i+1}=L^{-1}S\left(v_{i}\right)\rho_{i}$$

for i=0,…,m−1 where $L=I_{n}-k_{t}Q$ (Fig. 1). Consequently, the discrete model of the rOMT problem is given as follows:

(7) $$\underset{v}{min}\;F(v)=k_{s}k_{t}\rho^{T}M(v⊙v)+\beta {∥\rho_{m}-\rho_{1}^{obs}∥}^{2}$$

subject to:

(8a) $$\rho_{i+1}=L^{-1}S\left(v_{i}\right)\rho_{i},\;i=0,\ldots ,m-1,$$

(8b) $$\rho_{0}=\rho_{0}^{obs}.$$

One can prove that F(v) is quadratic in v and $S\left(v_{i}\right)$ is linear in $v_{i}$. Hence, following Steklova and Haber [27], the Gauss-Newton method is used to optimize for the numerical solution where the gradient $g(v)=\nabla_{v}F(v)=\frac{\partial F}{\partial v}$ and the Hessian matrix $H(v)=\frac{\partial^{2}F}{\partial v^{2}}$ are computed to solve the linear system Hx=−g for x in each iteration.

Next, we elaborate on the analytical derivation of g(v) and H(v). Noticing that in F(v), ρ and $\rho_{m}$ are dependent on v following the advection-diffusion constraint, we have

(9a) $$g=k_{s}k_{t}\nabla_{v}\left(\rho^{T}M(v⊙v)\right)+\beta \nabla_{v}\left({∥\rho_{m}-\rho_{1}^{obs}∥}^{2}\right)$$

(9b) $$=k_{s}k_{t}\left(2(M\;diag(v){)}^{T}\rho +{\left(\nabla_{v}\rho \right)}^{T}M(v⊙v)\right)+2\beta {\left(\nabla_{v}\rho_{m}\right)}^{T}\left(\rho_{m}-\rho_{1}^{obs}\right)$$

and

(10a) $$H=\frac{\partial g}{\partial v}\approx 2k_{s}k_{t}\rho^{T}\nabla_{v}(M\;diag(v))+2\beta {\left(\nabla_{v}\rho_{m}\right)}^{T}\nabla_{v}\rho_{m}$$

(10b) $$=2k_{s}k_{t\;}\mathrm{diag}\left(\rho^{T}M\right)+2\beta {\left(\nabla_{v}\rho_{m}\right)}^{T}\nabla_{v}\rho_{m},$$

where diag(.) is the function creating a diagonal matrix from the components of the given vector, and $\nabla_{v}$ is the operator of taking gradient with respect to v.

Considering the expressions of g and H, the difficulty lies in the computation of $\nabla_{v}\rho_{m}$ and $\nabla_{v}\rho$. Let $J≜\nabla_{v}\rho ={\left(J_{v_{j}}^{k}\right)}_{k,j}$ where $J_{v_{j}}^{k}=\frac{\partial \rho_{k}}{\partial v_{j}},$ k=1,…,m, j=0,…,m−1. From the constraint Eq. 8a, we have

(11) $$\rho_{k}=L^{-1}S\left(v_{k-1}\right)L^{-1}S\left(v_{k-2}\right)\ldots L^{-1}S\left(v_{0}\right)\rho_{0},\;k=1,\ldots ,m,$$

indicating that $\rho_{k}$ is only dependent on $v_{0},\ldots ,v_{k-1}$ but independent of $v_{k},\ldots ,v_{m}$, so that for ∀j≥k, $J_{v_{j}}^{k}=0$ holds. Therefore, J is a lower-triangular block matrix of the form

(12) $$J=\left(\begin{array}{cccc} J_{v_{0}}^{1} & & & \\ J_{v_{0}}^{2} & J_{v_{1}}^{2} & & \\ \vdots & \vdots & \ddots & \\ J_{v_{0}}^{m} & J_{v_{1}}^{m} & \cdots & J_{v_{m-1}}^{m} \end{array}\right)≜\left(\begin{array}{c} J_{1}\\ J_{2}\\ \vdots \\ J_{m} \end{array}\right),$$

where $J_{k}=\nabla_{v}\rho_{k}$ is the row block of J for k=1,…,m. If j<k,

(13) $$J_{v_{j}}^{k}=L^{-1}S\left(v_{k-1}\right)\cdots L^{-1}S\left(v_{j+1}\right)L^{-1}B\left(\rho_{j}\right),$$

where $B\left(\rho_{j}\right)=\frac{\partial }{\partial v_{j}}\left(S\left(v_{j}\right)\rho_{j}\right)$, which by the particle-in-cell method is linear in $v_{j}$ and dependent on the density $\rho_{j}$. Notice that the second term of the Hessian matrix H given above, involves computing the multiplication of two matrices of sizes 3mn×n and n×3mn, which is usually avoided in numerical implementation. Instead, we use a function handle that computes Hx in place of the coefficient matrix H so that the second term in Hx can be derived from twice the multiplication of a matrix and a vector. To sum up, we are going to compute

(14a) $$g=k_{s}k_{t}\left(2(M\;diag(v){)}^{T}\rho +J^{T}M(v⊙v)\right)+2\beta J_{m}^{T}\left(\rho_{m}-\rho_{1}^{obs}\right),$$

(14b) $$Hx=2k_{s}k_{t}\;diag\left(\rho^{T}M\right)x+2\beta J_{m}^{T}J_{m}x.$$

We can create two functions getJmx and getJmTy to compute $J_{m}x$ and $J_{m}^{T}y$ for any vector x and y, respectively. Within these two functions, $J_{m}x$ and $J_{m}^{T}y$ can be computed iteratively in observation of the recursive format of Eq. 13. Given that J is lower-triangular, $J^{T}y$ can be computed by recursively calling the function referred to as getJmTy. We can use nested function getJmTy(getJmx(⋅)) to get the second term of Hx. However, this algorithm spends the vast majority of time (more than 90%) solving the linear system Hx=−g with the MATLAB built-in function *pcg*. We therefore modify the previous algorithm to reduce the time spent on this computational bottleneck.

The major contributions of the present work are as follows:

1. We pre-compute all $S\left(v_{j}\right)$ and $B\left(\rho_{k}\right)$ for k=1,…,m, j=0,…,m−1 and make them inputs when calling functions getJmx and getJmTy to eliminate unnecessary redundant computations of advection-related matrices.
2. We combine nested function getJmTy(getJmx(·)) into one function in light of the poor performance of transferring function handles in nested functions.
3. We add the option of running the rOMT model with multiple input images $\rho_{0}^{obs},\;\rho_{1}^{obs},\ldots ,\rho_{p-1}^{obs}$ where p>2 in parallel to further reduce runtime.

Consequently, we found a significant improvement in the efficiency of solving the linear system. See Algorithm 1 for the detailed process.

**Algorithm 1:** Gauss-Newton Method

Load in $\rho_{0}^{obs}$, $\rho_{1}^{obs}$
v= initial guess
**for** i=1,2,…,MaxIter **do**
Compute interpolations $\rho =AdvDiff\left(\rho_{0}^{obs},\;v\right)$
Compute $S\left(v_{j}\right)$ and $B\left(\rho_{k}\right)$ for j=0,…,m−1, k=1,…,m
Compute gradient g and Hessian matrix function handle Hx
Solve linear system Hx=−g for x
Do line search to find length l
**if** line search fails **then**
**return** v
**end if**
Update v=v+lx
**end for**
**return** v

### 2.2 Lagrangian Coordinates

Instead of representing the system employing Eulerian coordinates, one can get a Lagrangian representation of the above framework in the standard way. This is of course very useful for tracking the trajectories of particles and for investigating the characteristic patterns of fluid dynamics. This Lagrangian method has been used as a visualization method in [6, 10, 18].

Briefly, the method begins with defining the augmented velocity field $\tilde{v}=v-\sigma \nabla \;log\;\rho$ and putting it into the advection–diffusion equation to get a zero on the right-hand side

(15) $$\frac{\partial \rho }{\partial t}+\nabla \cdot (\rho \tilde{v})=\sigma \Delta \rho -\sigma \nabla \cdot (\nabla \;log\;\rho )=0$$

which gives a conservation form of the advection–diffusion equation Eq. 2a. We apply Lagrangian coordinates L(t,x) such that

(16a) $$\frac{\partial L}{\partial t}=\tilde{v}(t,L(t,x))$$

(16b) $$L\left(0,x\right)=x,$$

to track the *pathlines* (i.e. trajectories) of particles with the starting coordinates at t=0 (Eq. 16b) and the time-varying augmented velocity field $\tilde{v}$. Along each binary pathline, the speed s=||v|| may be calculated at each discrete time step, forming a pathline endowed with speed information which we call a *speed-line*. The speed-lines indicate the relative speed of the flow over time.

This representation provides a neat way of computing certain dimensionless constants that are very popular in CFD, in particular, the Péclet (Pe) number. It has been used by several groups [15, 23] in neuroscience to study the motion of cerebrospinal fluid (CSF) within the brain. Of special importance is the determination of regions where advection dominates or where diffusion dominates. In our model, we define Pe number as follows:

(17) $$Pe=\frac{∥v∥}{\sigma ∥\nabla \;log\;\rho ∥}.$$

This measures the ratio of advection and diffusion. Similar to speed-lines, we can compute and endow Pe along the binary pathlines to form the *Péclet-lines*.

In order to visualize in 3D rendering, we interpolate the speed-lines and Péclet-lines into the original grid size by taking the averages of the endowed speed and Pe values within the same nearest voxel to derive the smoothed speedmap and Pemap, respectively. Additionally, the directional information of the fluid flow is thereby captured by connecting the start and end points of pathlines, obtaining vectors which we refer as *velocity flux vectors*. Even though they may lose intermediate path information compared to pathlines, these vectors can provide a clearer visualization of the movement.

## 3 Results

This section comes in three parts. In the first two parts, we test our methodology on a synthetic geometric dataset, and then a real DCE-MRI rat brain dataset. We show the visualized results in both cases. Finally, we compare the computational performance of the upgraded rOMT algorithm with that of the previous one [6, 10, 18] on the two forementioned datasets and report a significant saving in runtime.

### 3.1 Gaussian Spheres

**Fig. 2:**
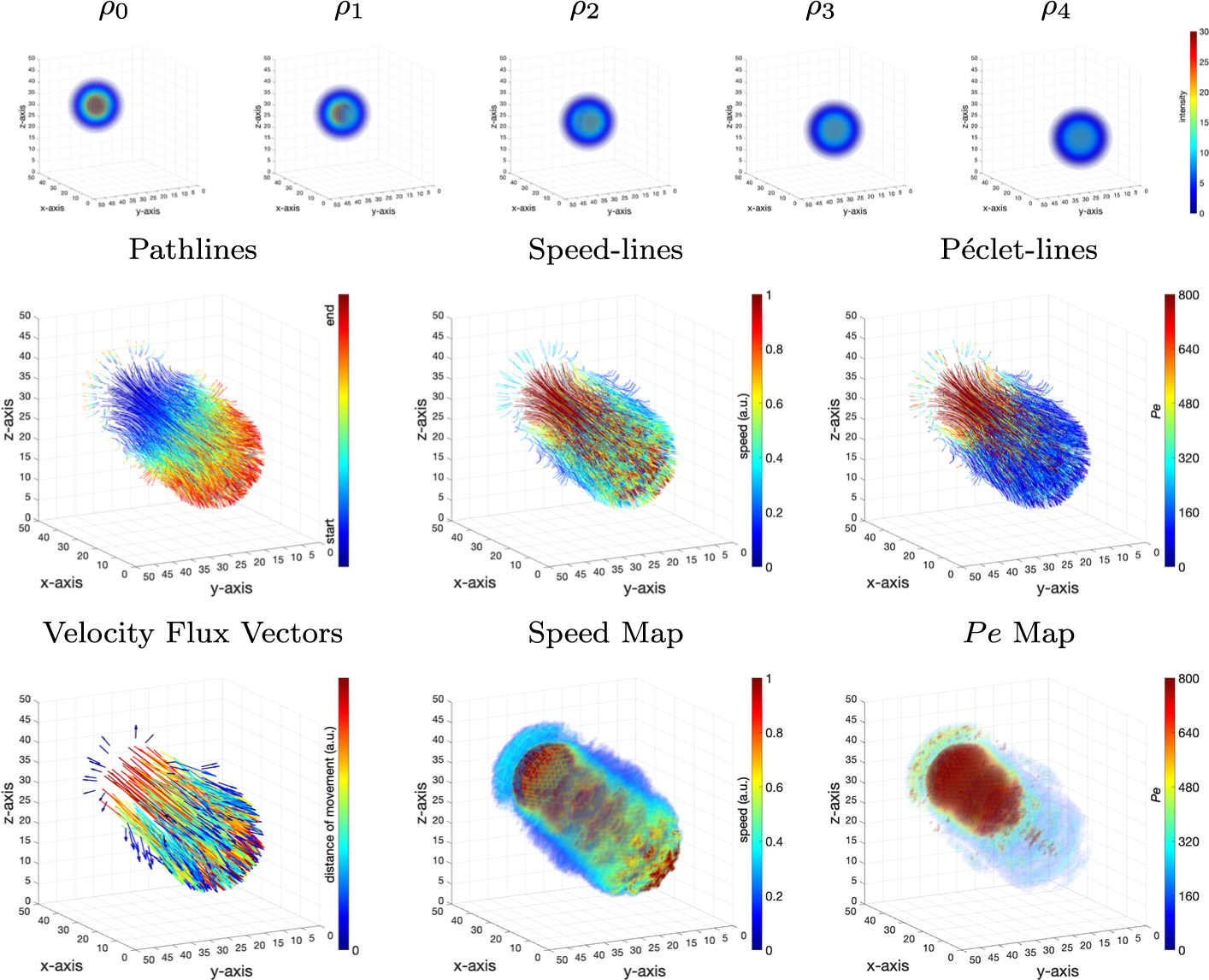
3D Geometric Gaussian Spheres Dataset: First row: a series of synthetic Gaussian spheres as inputs to the rOMT model. The sphere is advectively moving forward while simultaneously diffusing locally. Second and third rows: illustrative outputs from the Lagrangian rOMT methodology to visualize the fluid dynamics. Pathlines, color-coded with start and end times, show the trajectories of particle movement. The speed-lines show the relative speed at the corresonding location along pathlines. The Péclet-lines indicate the local transport motion of advection-dominated (higher value) or diffusion-dominated (lower value) along pathlines. The speed map and the Pe map shown in 3D rendering are smoothed interpolations of speed-lines and Péclet-lines on the numerical grid, respectively. Velocity flux vectors are vectors obtained by connecting the initial and terminal points of the pathlines, which are color-coded with the length of vectors, illustrating the overall direction of the movement. These outputs show that the higher speed distributes mainly in the core of the Gaussian sphere and that the transport is first dominated by advection but quickly diffusion prevails

Five 3D Gaussian spheres of image size 50 × 50 × 50 were created, $\rho_{0},\ldots ,\rho_{4}$, as successive input images fed into the rOMT algorithm and its Lagrangian post-processing (Fig. 2, first row). The initial mass distribution $\rho_{0}$ is a 3D dense Gaussian sphere, and it moves forward (advection) with mass gradually diffusing into the surrounding region over time. To be specific, $\rho_{i}$ was created from a *3D* Gaussian function

(18) $$G_{i}(x,\;y,\;z)=\frac{100}{\sqrt{2\pi }}exp\left(-\frac{(x\;-\;0.8i{)}^{2}+(y\;-\;0.8i{)}^{2}+(z\;-\;0.8i{)}^{2}}{2}\right)$$

for i=0,...,4, which was later discretized by taking uniform step length $=\frac{12}{49}$ and fit into the same 50 × 50 × 50 numerical grid. Advection was therefore naturally applied due to the movement of the Gaussian centers. Diffusion was further added to $\rho_{i}$ by applying a 3D Gaussian filter to $\rho_{i}$ with standard deviation $=(i+1)\sqrt{0.2}$ for i=1,…,4. The unparalleled runtime for this dataset was about 26min on a 2.6 GHz Intel Core i7–9750H, 16 GB RAM, running macOS Mojave (version 10.14.6) with MATLAB 2019b. Please refer to Table 1 for parameters used in the experiment.

The resulting interpolated images from the rOMT algorithm are made into a video on Github. Other returned outputs are illustrated in Fig. 2. The binary pathlines, which are color-coded with the numerical start and end time, show the trajectories of particles. The resulting velocity flux vectors point in the direction of the movement and the color code shows relatively how far a particle is transported during the whole process. The speed-lines and the interpolated speed map indicate that the core of the Gaussian spheres are of higher speed compared to the outer regions. The Péclet-lines and Pe map show that in the early stage of the transport, the motion is mainly advective in nature. However, in the later time, diffusion takes over. This change of dominated motion is within expectation in that dissolvable substances always have the tendency to eventually be equally mixed with the solution as a result of diffusion, regardless of the existence of an imposed velocity field.

### 3.2 DCE-MRI Rat Brain

To further test our method on real-world data, we ran our algorithm on a DCE-MRI dataset consisting of 55 rat brains. During the MRI acquisition, all the rats were anesthetized and an amount of tracer, gadoteric acid, was injected into the CSF from the neck, moving towards the brain. The DCE-MRI data were collected every 5min and were further processed to derive the % signal change from the baseline. Data for each rat contained 23 images of size 100 × 106 × 100, spanning a 110-min time period. We put every other image (in total 12 images) within a masked region into our Lagrangian rOMT method to reduce the computational burden. To avoid constantly introducing new data noise into the model, we utilize the final interpolated image from the previous loop as the initial image of the next loop. The computation of the rOMT model was performed consecutively using MATLAB 2018a on the Seawulf CPU cluster using 12 threads of a Xeon Gold 6148 CPU, which took about 4h for each rat. The Lagrangian post-processing method took between 2 and 3min for each case. Please refer to Table 1 for parameters used in this experiment.

**Fig. 3:**
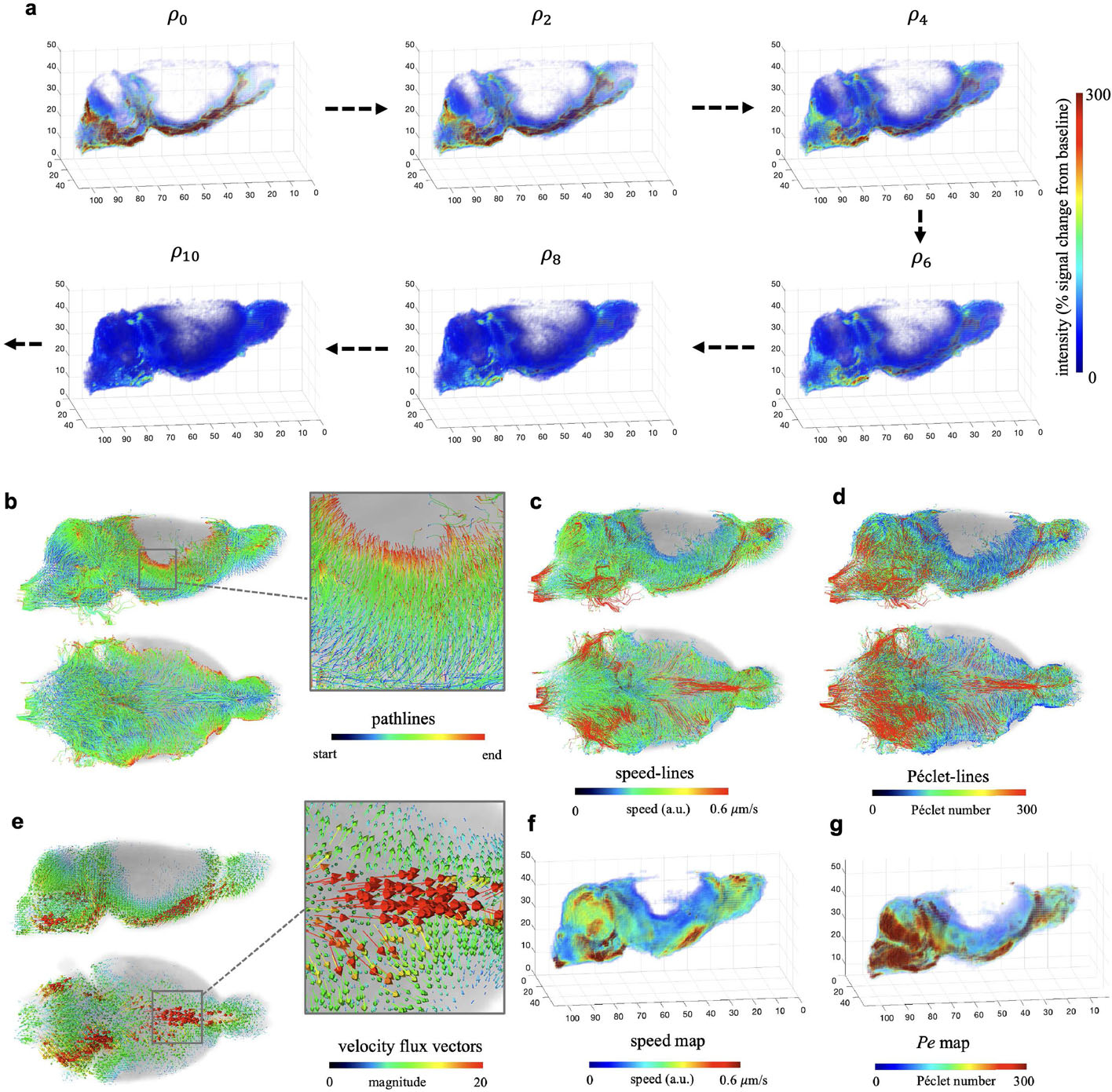
3D DCE-MRI Rat Brain Dataset: **a** The input data is 12 successive images, $\rho_{0},\;\ldots ,\;\rho_{11}$, selectively shown in 3D rendering. **b**–**g** The outputs from the Lagrangian rOMT methodology. The pathlines give the trajectories of tracer over the 110-minute period. The velocity flux vectors, color-coded with the length of vectors and shown scaled by 0.25, indicate that in addition to penetrating into the brain parenchyma, there are strong flows moving within CSF towards the nose. According to the speed-lines and speed map, higher speed occurred mainly along the large vessels and quickly slowed down after entering the brain. The Péclet-lines and Pe map identified the transport along vessels and in CSF mainly as advection-dominated by the relative higher Pe numbers therein. In contrast, the movement motion was dominated by diffusion after the entry of tracer into deeper brain

In Fig. 3, we display the data and results of an example 3-month-old rat. As the pathlines and velocity flux vectors illustrate, the tracer partly entered the brain parenchyma via the CSF sink and was partly drained out towards the nose. From the speed-lines and speed map, the higher speed occurred mainly along the large vessels, which is also recognized as advection-dominated transport according to the Péclet-lines and Pe map. When the tracer entered the brain parenchyma, the movement motion was mainly dominated by diffusion due to the relatively low values there.

To further demonstrate that our methodology is useful in tracking and visualizing the fluid flows in rat brains, we plotted and made a video of the pathlines, speed-lines and Péclet-lines in 3D from a same 12-month-old rat, which is available on Github.

### 3.3 Computational Performance

As detailed in Sect. 2.1, we improved the current rOMT code by eliminating repeated computation in nested functions and by optimization of the algorithm. An important part of this reduction in computational time was done by pre-computing intermediate results of the advective steps that were used either throughout the calculations or for a specific step. We compared the upgraded algorithm with the previous algorithm [6, 10, 18] by recording the runtime of rOMT code on the Gaussian sphere dataset at scaled image size (N = 6, each of 4 loops) in Sect. 3.1 and the DCE-MRI dataset in Sect. 3.2 (N = 55, each of 11 loops). We also compare the algorithms in term of accuracy on the Gaussian sphere dataset.

Note that the runtime here refers to the time it takes from loading in the data to saving the returned outputs so that the pre-computation is included into the runtime for the upgraded algorithm.

To analyze the time complexity and accuracy of the algorithm, we considered the original Gaussian sphere images of size $N^{3}=50^{3}$ and scaled N by a factor of 0.5, 0.75, 1, 1.25, 1.5 and 1.75 to obtain spheres of sizes 25^3^, 38^3^, 50^3^, 63^3^, 75^3^ and 88^3^, respectively. We put the six groups of data into the rOMT code using MATLAB 2019a on the Seawulf CPU cluster with 12 threads of a E5–2683v3 CPU.

**Fig. 4:**
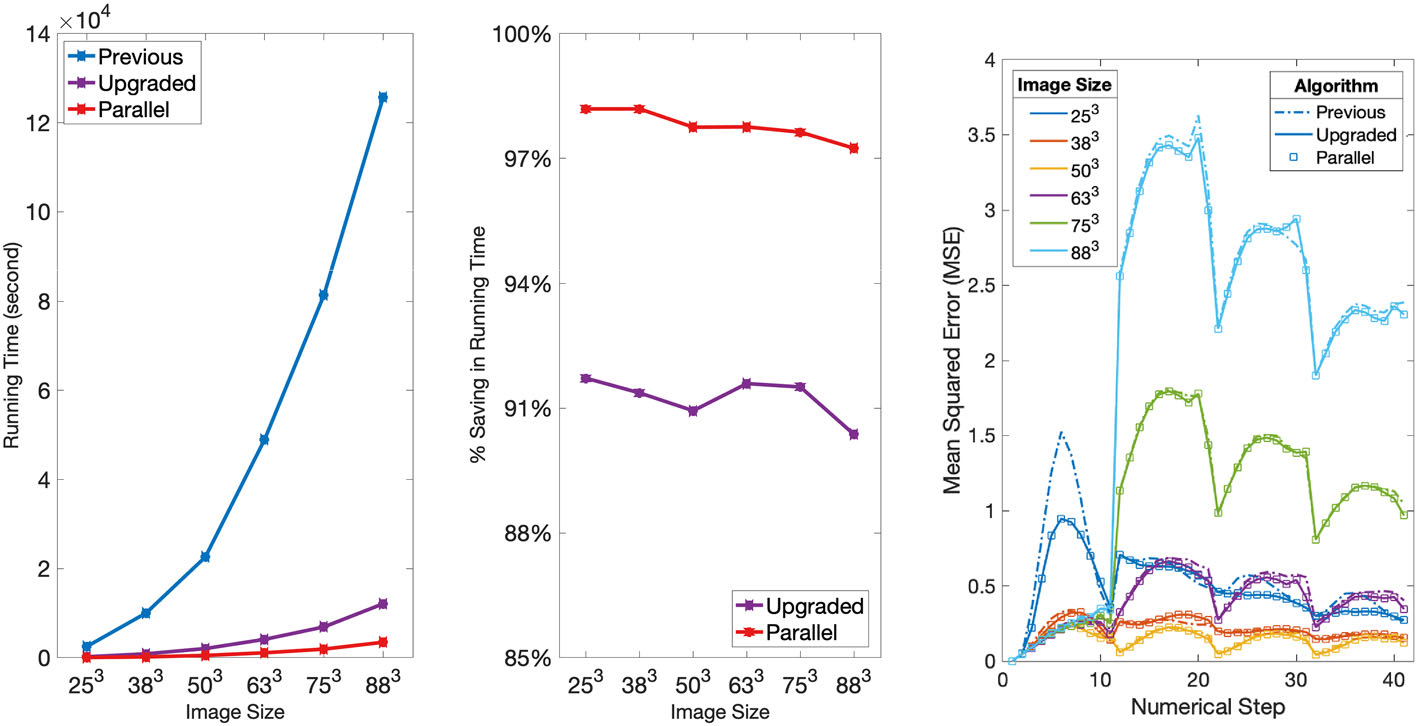
Comparison of computational performance on the Gaussian sphere dataset: Left: the comparison of runtime of previous, upgraded and upgraded+parallel code at scaled image size. Middle: The percent of saving in runtime compared with the previous code at scaled image size. The upgraded code realized 91.25% ± 0.51% reduction and can be further improved to 97.79% ± 0.36% if run in parallel. Right: the mean squared error (MSE) curves of three versions of code on scaled image sizes over the numerical steps

As illustrated in Fig. 4 (left and middle), the runtime increases drastically as N linearly scales up, especially for the previous code. For example, the previous code took 0.70h to run on the 25^3^ size input, 13.61h on the 63^3^ size input, and 1 day and 10.91h on the 88^3^ size input. The upgraded code without parallelization greatly reduced the runtime to 3min, 1.14h, and 3.36h, respectively. By analyzing the six groups of data statistically, we found that in general a 91.25% ± 0.51% reduction and a 97.79% ± 0.36% reduction in runtime were realized by the upgraded code and the parallelized code, respectively, compared with the previous version.

Now we define the *accuracy* of the rOMT algorithm. Note that rOMT is a data-driven method that tries to find the most likely evolving process between two images. It means that it will be difficult to define the ground truth which we can compare with. However, in the Gaussian sphere dataset, we can approximate the most likely ground truth, the intermediate images, motivated by the way we create these images. Recall that the input images were synthesized by adding a linearly increasing shift to the center of the Gaussian function (advection) and applying a Gaussian filter with a linearly increasing standard deviation (diffusion). From $\rho_{i}$ to $\rho_{i+1}$, rOMT produces m intermediate interpolations with the last one matching $\rho_{i+1}$. We obtain the approximated intermediate ground truth images $\rho_{i,j}^{\mathrm{obs}}$ by applying a Gaussian filter with standard deviation $=\left(i+\frac{j}{m}+1\right)\sqrt{0.2}$ to a Gaussian function

(19) $$\frac{100}{\sqrt{2\pi }}exp\left(-\frac{{\left(x\;-\;0.8\left(i\;+\;\frac{j}{m}\right)\right)}^{2}\;+\;{\left(y\;-\;0.8\left(i\;+\;\frac{j}{m}\right)\right)}^{2}\;+\;{\left(z\;-\;0.8\left(i\;+\;\frac{j}{m}\right)\right)}^{2}}{2}\right)$$

for i=0,...,3 and j=1,...,m−1. We then calculate the mean squared error (MSE) of each pair of approximated ground truth image and the interpolated image from rOMT as a measurement of the accuracy of the algorithm. The lower the MSE is, the more accurate the algorithm models the dynamics. The MSE curves throughout the numerical steps on scaled images and three versions of the algorithm are demonstrated in Fig. 4 (right). By comparing different input image sizes, the rOMT algorithms gave lowest MSE (0.1412 ± 0.0577) and therefore the highest accuracy over time in the original images of size 50^3^. It indicates that the parameters used in Table 1 are best tuned for image size around 50^3^. It is notable that when images are scaled up too much, both the runtime and MSE will rocket, perhaps implying an image size limit to the rOMT method. By comparing the three versions of algorithm, we do not observe any difference in MSE between the upgraded one and the parallelized one over time. We notice some differences in MSE between the previous one and the upgraded one. However, the differences are very minor, especially for the original image size.

**Fig. 5:**
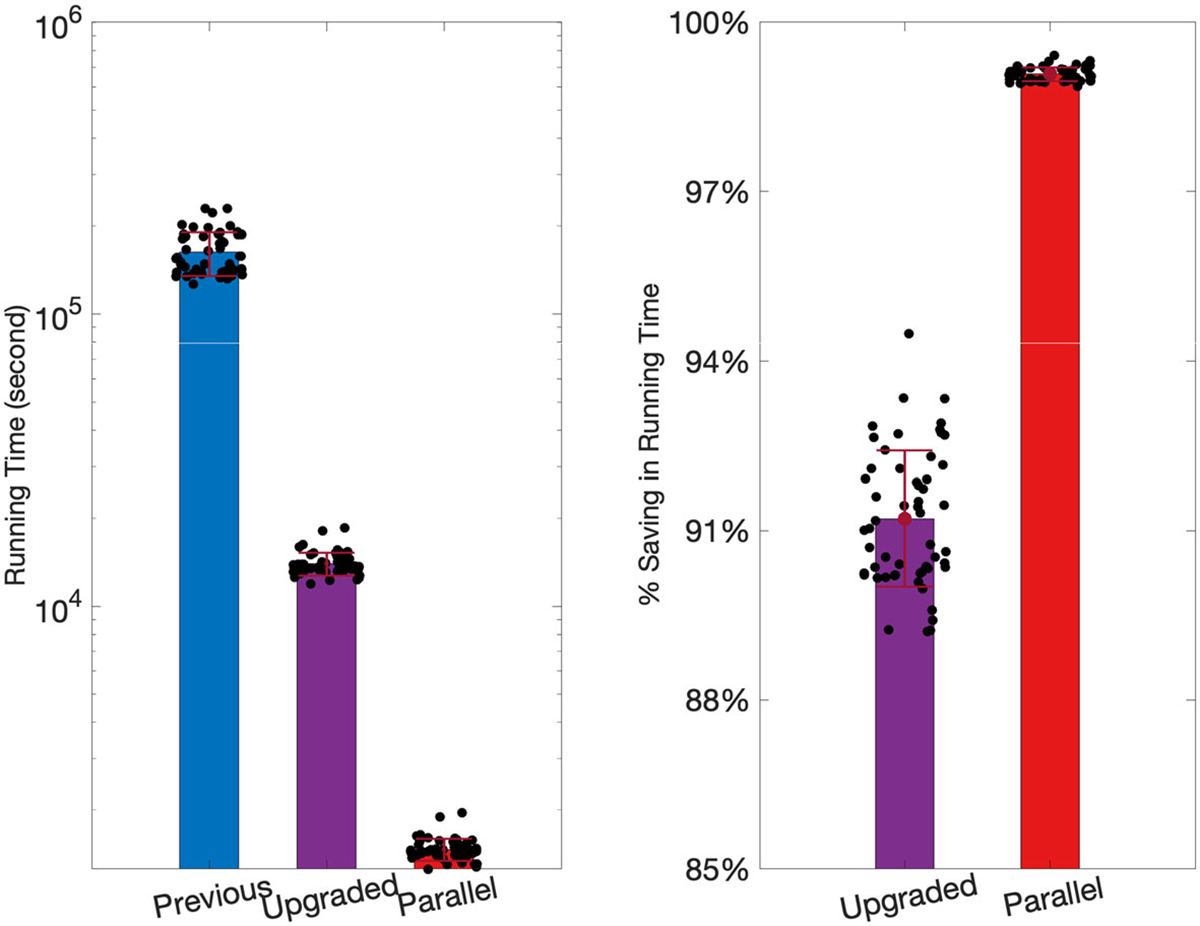
Comparison of runtime on the rat brain dataset: Left: the comparison of runtime in logarithmic scale of previous (45.18 ± 7.64h), upgraded (3.89 ± 0.35h) and upgraded+parallel (0.41 ± 0.03h) code. There are in total 55 data points and each case consists of 12 input images resulting in 11 loops. Right: The percent of saving in runtime compared with the previous code. The upgraded code realized 91.21%± 0.12% reduction and can be further improved to 99.08% ± 1.21% if run in parallel

For the rat brain dataset, the previous algorithm took 45.18 (± 7.64) hours to run a case. However, it took only 3.89 (± 0.35) hours for the upgraded algorithm to run and 0.41 (± 0.03) hours if run in parallel, resulting in a significant reduction in runtime by 91.21% (± 1.21%) and 99.08% (±0.12%), respectively (Fig. 5).The runtime depends on various factors, such as the size of input image, the number of input images p, the diffusive coefficient σ, the number of time intervals m, *etc*. However, this significant improvement of efficiency is believed to be comprehensive as all parameters were kept fixed for the comparison.

## 4 Discussion

Our rOMT methodology, together with its Lagrangian post-processing approach, models the dynamic fluid flows based on the advection–diffusion equation and the theory of OMT. This method is largely data-driven, meaning that there is no ground truth at hand to compare with especially when it comes to real-life image data. Taking σ=0 in our model, the square root of the obtained infimum in the cost function Eq. 1 gives $L^{2}$ *Wasserstein metric*, which has vast applications to many fields [29, 30].

It is interesting to note that rOMT is mathematically equivalent to the Schrödinger bridge [19,20], and thus the proposed algorithm may prove useful for a number of problems in which this mathematical model is relevant. We should note that while the Schrödinger bridge is formally similar to OMT (σ=0), it has a substantially different motivation and interpretation. OMT was originally formulated in an engineering framework as the problem to optimally transport resources between sources and destinations. Erwin Schrödinger’s motivation for the Schrödinger bridge was based on physics and the so-called “hot gas experiment” that led to a certain maximal likelihood problem. The aim was to link quantum theory to classical diffusion processes. In both cases (OMT and the Schrödinger bridge), one starts with two probability measures. In OMT, the measures are regarded as the initial and final configurations of ***resources whose transportation cost has to be minimized*** among all possible couplings: this is the Kantorovich formulation [29, 30]. In comparison, the measures employed in the Schrödinger bridge represent initial and final probability ***distributions of diffusive particles***, and one searches for the most likely evolution from one to the other. It may be regarded as an entropy minimization problem in path space, and gives a natural data-driven model for a number of dynamical processes arising in both physics and biology, as described above in our models of fluid flow in the brain. The Sinkhorn algorithm is also widely used to solve an entropy-regularized OMT problem [9]. But it is put into an engineer setting to best reallocate resources and lacks a flavor in fluid dynamics to model the fluid flows in brain. In contrast, our rOMT method involves solving the important advection–diffusion equation in fluid dynamics which guides the whole transport process, and the returned dynamic velocity fields can help measure and visualize the trend of the fluid flows.

**Fig. 6:**
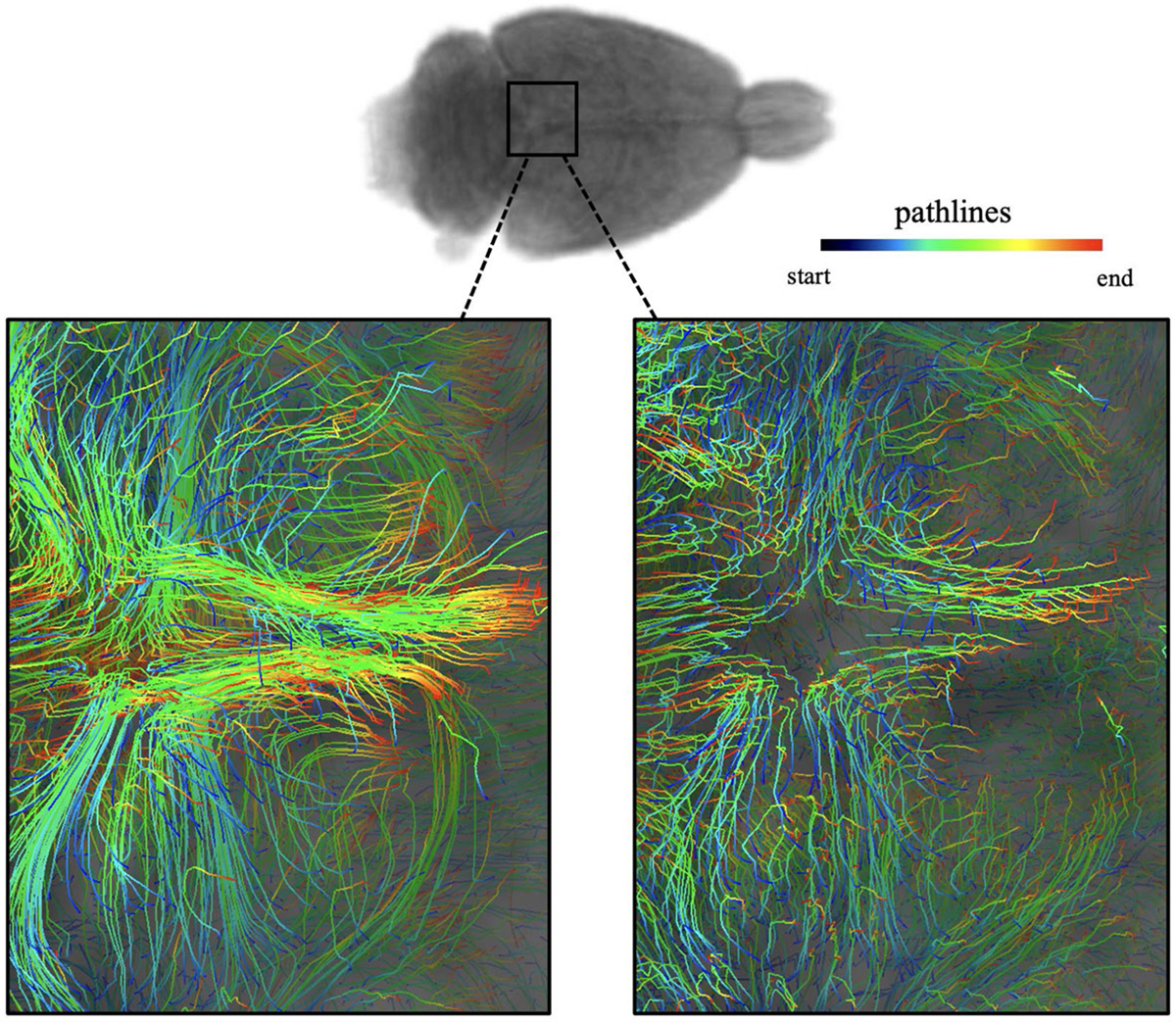
Comparison of unparallelized and parallelized algorithms on pathlines: The pathlines of an example rat brain viewed from the top. Left: pathlines from continuously feeding final interpolated image into next loop. Right: pathlines from parallelized computation. The left ones are smoother and more in shape of clusters. The right ones show some fluctuations in the obtained pathlines due to constantly introducing new data noise into the system, but the overall direction of movement is aligned with the left. The left requires about 10-fold runtime of that on the right

Numerical algorithms based on finite difference scheme and optimization can be very time-consuming on large 3D images. We realized a remarkable reduction of runtime of the rOMT algorithm, cutting a two-day running time down to 4h and even less if run in parallel. One may notice that there can be some defects in the connection of the velocity fields when applying the rOMT algorithm independently to consecutive images in a given series run in parallel. For example, in Fig. 2 the speed map shows discontinued boundaries due to four independent loops. This can be ameliorated by feeding the final interpolated image from the previous loop into the next one as the initial image to give smoother velocity fields, as we did for the DCE-MRI rat brain dataset. However, by doing so parallelization is out of question because the loops are connected in time. The longer running time comes with the advantage of much smoother pathlines for meaningful visualization (Fig. 6). One must be wise weighting between a quicker algorithm and smoother velocity fields. If the continuity and smoothness of velocity fields is highly emphasized, a multi-marginal model should be considered and longer time to run is also expected. In addition, in Sect. 2.1 we mentioned that numerically we employed a free end-point version of rOMT (Eqs. 3–4b) when implementing the rOMT model (Eqs. 1–2b) on the noisy DCE-MRI data. The motivation was to avoid bringing in new data noise resulting from forcing a fixed end-point condition. Now from the comparison shown in Fig. 6, it seems that the image noise does indeed affect the results even with a free end-point model. In the future, some work should be done in image pre-processing to remove the data noise beforehand.

In our rOMT model, a diffusion term σΔρ is added to the continuity equation to make it an advection–diffusion equation of a type used in fluid dynamics. The added term does not affect the total mass conservation constraint $\int_{\Omega }\;\rho_{0}(x)dx=\int_{\Omega }\;\rho_{1}(x)dx$, because diffusion obeys the law of conservation of mass. In fact, we can rewrite the advection–diffusion equation in conservation form (Eq. 15) where this is explicitly seen. We would like to emphasize that adding this diffusion term is essential, because theoretically the phenomenon of diffusion exists wherever there is a non-zero gradient of the solute concentration in the fluid. Despite efforts in understanding fluid motions within the brain, the question of whether diffusion or advection drives or dominates the fluid flows still remains controversial [3,22]. That is the reason why we added a diffusion term into the model and also why we introduced a measurement, the Péclet number (Eq. 17), to reflect the relative intensity of advection against diffusion. Without the diffusion term, the Péclet number is simply infinity (advection) everywhere, which makes the comparison of advection vs. diffusion impossible. From the perspective of studying fluid flows in brain, our introduction of the diffusion term makes biological sense, since as shown in Fig. 3d and g, higher Péclet numbers occur mainly along the large vessels (advection) and lower values are mainly in the brain tissue (diffusion), which is in agreement with the present understanding in neuroscience. From the algorithmic perspective, adding a diffusion term will result in a longer runtime than the regular OMT. The overall trend is that the higher the diffusion coefficient is, the longer it takes to run the algorithm. However, this additional computational “burden” is understandable due to the necessity of adding a diffusion term for the applications in neuronscience. Moreover, notice that in our formulation, a constant diffusion coefficient σ>0 is used to approximate the transport behavior of diffusion in the fluid environment of brain. However, despite the successful application of rOMT to neuronscience, realistically the diffusion should be spatially dependent or even time dependent in the brain. There has been work using a non-linear diffusion term that takes the edge information into consideration [32].

An unbalanced version of the rOMT model has also been proposed where an independent source term is added into the formulation to help better model and visualize the fluid flows in the brain [5]. With this source term, mass can be created or destroyed instantaneously, so the mass conservation constraint of the initial and final density/mass distributions can be removed. The numerical method in [5, 32] is largely based on the upgraded algorithm described here in which pre-computation is also used to cut down upon the total runtime.

## 5 Conclusions

We introduced the rOMT and Lagrangian post-processing methodology as an efficient algorithm to track and visualize fluid flows, and in particular to track the trajectories of substances in the glymphatic system using DCE-MRIs from a CFD perspective. Quantitative measurements, speed and the Péclet number are also provided along with the pathways to help uncover features of the fluid flows. We improved the previous code [6, 10, 18] by removing redundant computation and significantly saved the runtime by 91% without impairing the accuracy, and we offer the option to further reduce the runtime by running the algorithm in parallel.

## Figures and Tables

**Table 1:** Parameters used in rOMT algorithm

Parameter	Definition	Value for geometric data	Value for brain data

$n_{1}$	Grid size in x axis	50	56
$n_{2}$	Grid size in y axis	50	106
$n_{3}$	Grid size in z axis	50	51
p	Number of input images	5	12
m	Number of time intervals	10	
$k_{t}$	Length of each time interval	0.4	
$k_{s}$	Length of spatial grid	1	
σ	Diffusion coefficient	0.002	
β	Weighting parameter in cost functional	5000	

## Data Availability

The code for the rOMT algorithm and Lagrangian post-processing is available in https://github.com/xinan-nancy-chen/rOMT_spdup, which also contains the synthesized Gaussian sphere dataset and two sample data of the DCE-MRI rat brain dataset. The full rat brain dataset is available upon reasonable requests. The data of runtime comparison is available in https://github.com/xinan-nancy-chen/rOMT_spdup/blob/main/Runtime.xls. The data of MSE comparison is available in https://github.com/xinannancy-chen/rOMT_spdup/blob/main/MSE.xls.
